# Supplementary material for: Atomic scale volume and grain boundary diffusion elucidated by in situ STEM
Source: Nat Commun. 2023 Nov 22;14:7601. doi: 10.1038/s41467-023-43103-7 (PMC10663537; doi:10.1038/s41467-023-43103-7)
Supplement: Supplementary file 1 — Supplementary Information [file 41467_2023_43103_MOESM1_ESM.pdf]

Supplementary Information

Atomic Scale Volume and Grain Boundary Diffusion elucidated by *in situ* STEM

Schweizer et al.

Supplementary Figures

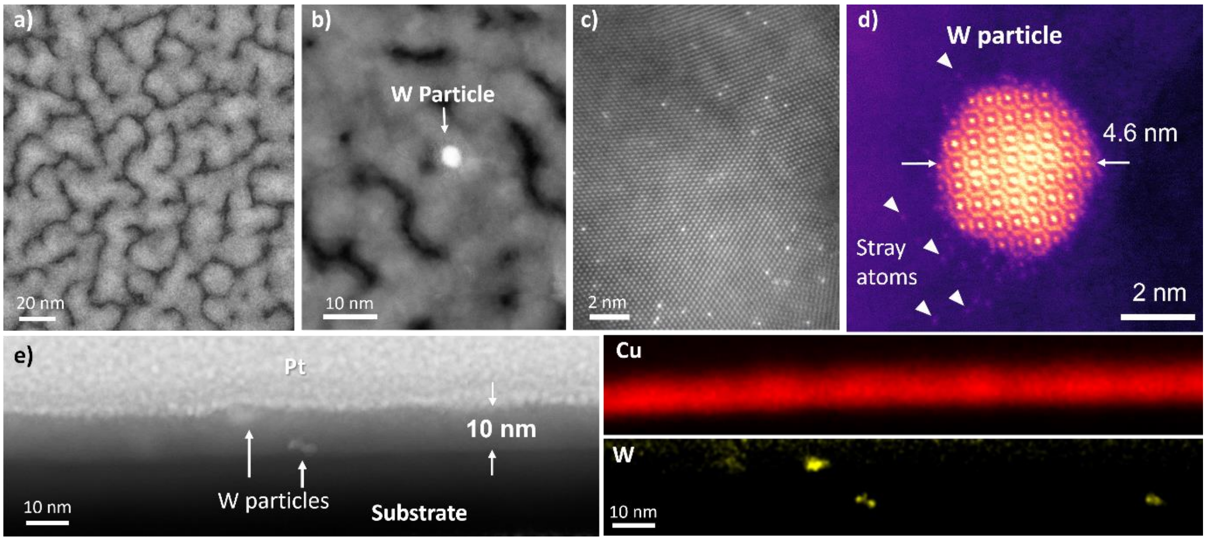

Supplementary Figure 1: Overview of deposited thin films used in this study. a) Medium magnification image of a deposited Al thin film showing a typical discontinuous structure. b) Region of the same sample containing a particle from the co-sputtering process with a nanoparticle gun. c) Diluted impurities embedded in the film at atomic resolution. Counting the number of impurity atoms and comparing that to the observed sample volume we get an impurity concentration of around 150 ppm. d) Single tungsten particle embedded in the film with a cloud of stray atoms around it. e) Cross-section of a copper thin film with tungsten particles and impurities and corresponding EDX maps. The thickness in this sample was in the range of 10 nm.

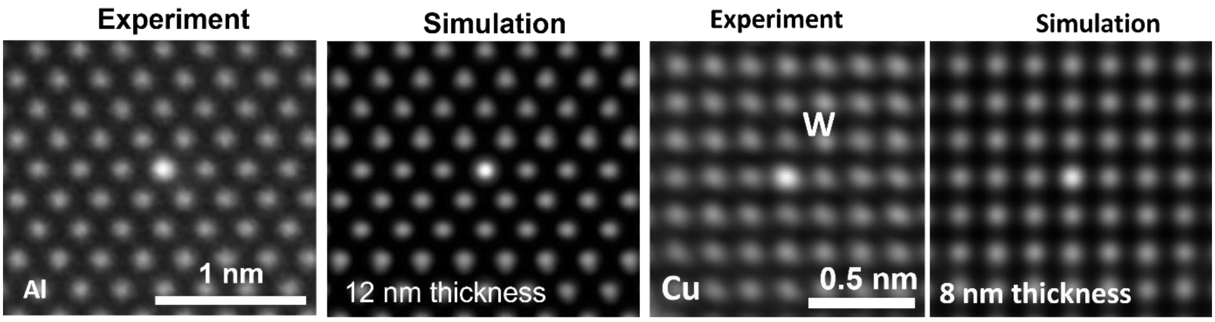

Supplementary Figure 2: HAADF STEM image simulations of isolated impurity atoms in a metal matrix and comparison to experimental images. Left: Al crystal in 110 orientation with a simulated thickness of 12 nm. Right: Cu crystal in a 100 orientation with a thickness of 8 nm. For the simulation a supercell of the host element was created and then a single atom near the centre of the cell replaced by a tungsten impurity. Image simulations were performed using Prismatic.

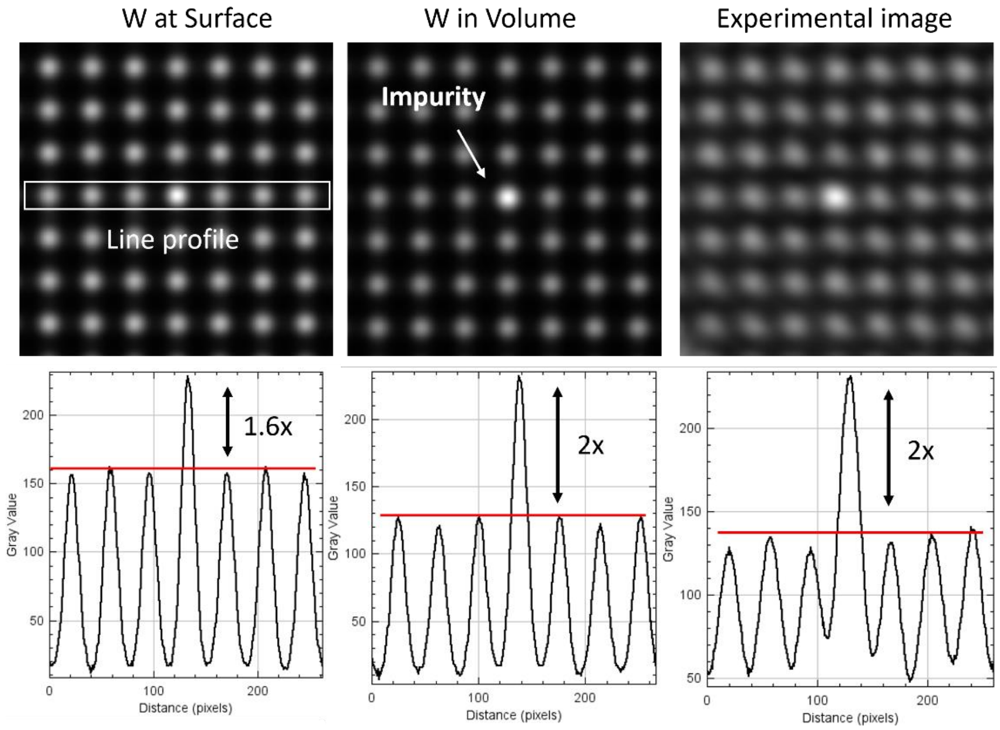

Supplementary Figure 3: Simulations comparing surface and volume sites. The simulation cell has a thickness of 8 nm and has a single Cu atom replaced by a W atom. In the first case this atom is placed at the surface of the material whereas in the second case it is placed in the center of the volume. The relative intensity of atomic columns with and without impurity is compared in a line profile, with a surface atom having a lower relative signal than the volume atom. Comparing these simulations to an experimental image the relative contrast matches that of a volume atom. While these simulations are non-exhaustive, they are a good qualitative indication of the depth of impurity atoms.

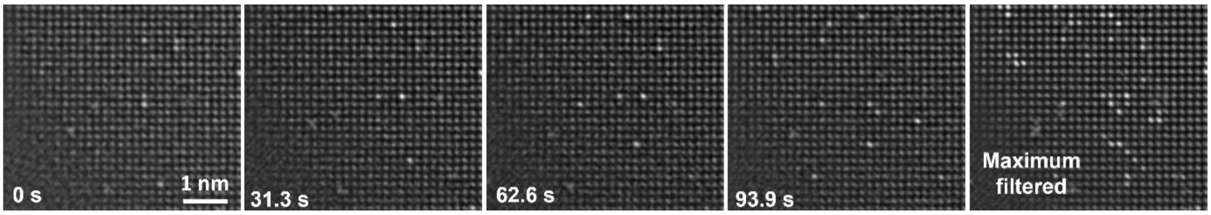

Supplementary Figure 4: Excerpt of the image series corresponding to Figure 1. Images acquired at 385°C with a raw frametime of 0.797 s. Each frame displayed is made up of an average of 4 raw frames. The images have been scaled by a factor of 2 and bandpass filtered. The final image is obtained by maximum filtering the entire image series. There is additional contrast not located on atomic columns in the bottom left of the images which may originate from W atoms on sessile surface spots are on grain boundary sites.

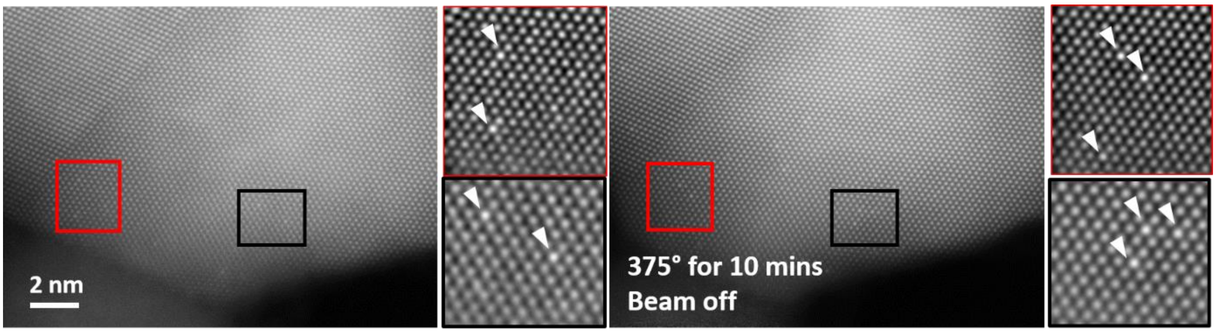

Supplementary Figure 5: Diffusion while the electron beam is switched off. a) STEM image of impurities in copper and two cropped areas showing the positions of selected atoms. b) STEM image of the same area after the sample has been annealed at 375° C for 10 mins with the electron beam switched off and the same cropped regions. The arrangement of the atoms has clearly changed, indicating a thermally driven diffusion process taking place without the electron beam. Compared to in situ observations, the atoms cannot be directly correlated as the path in between the two states is unknown.

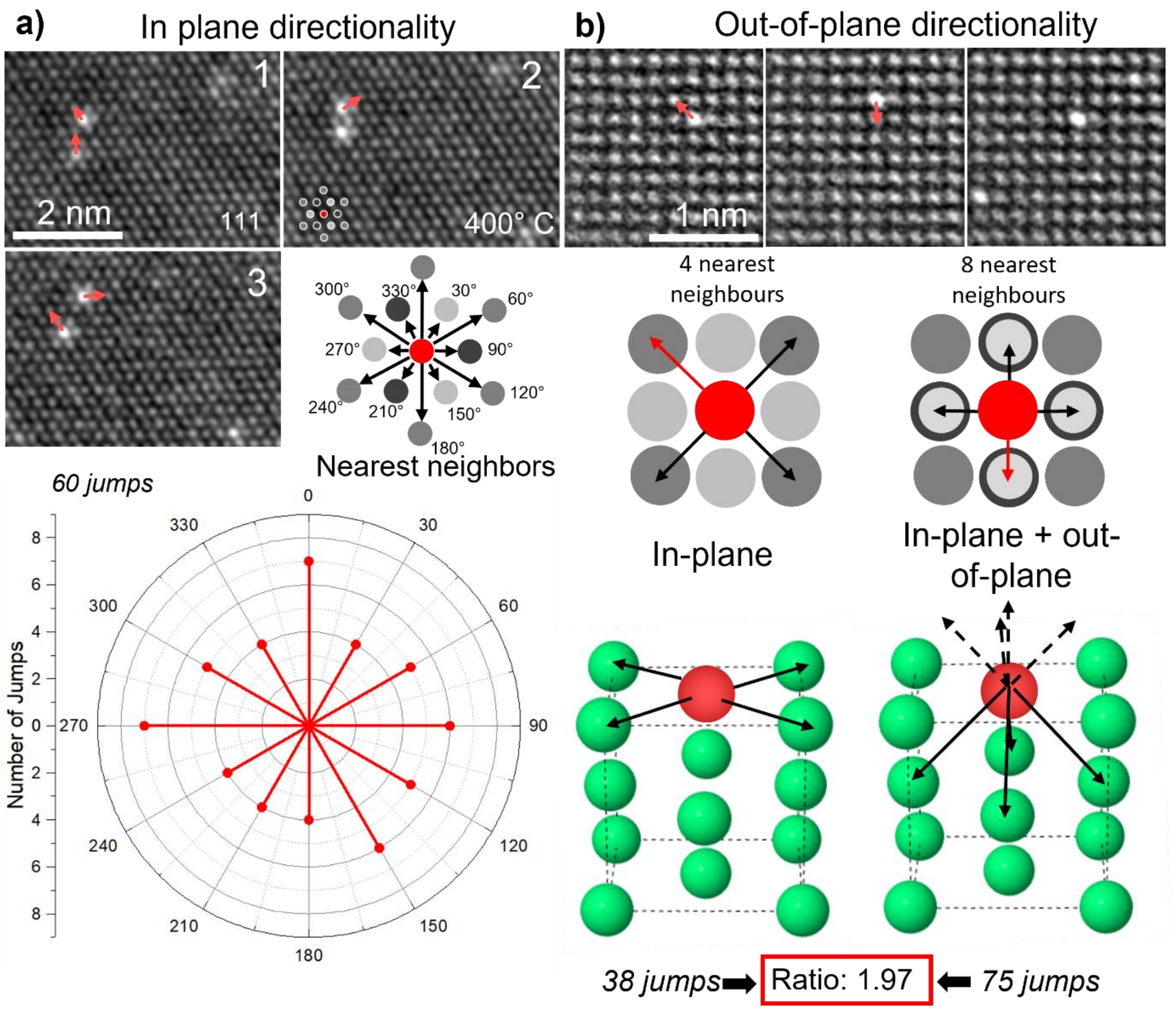

Supplementary Figure 6: Directionality evaluation of diffusive motion. a) Diffusion at 400°C viewed in a 111 projection. The direction of 60 jumps were classified according to their direction. Within the limited statistics a random direction of motion was seen. b) Quantification of the ratio of in-plane to out-of-plane jumps. In the 100 projection 4 nearest neighbour jumps are entirely contained within the plane of observation whereas 8 nearest neighbour jumps have in-plane and out-of-plane components. If the motion of the atoms is random this ratio should be reflected in the jumps. Quantifying 113

jumps yields a ratio close to 4:8 signifying a random motion. For surface diffusion to a nearest neighbour site the entire trajectory is contained in the plane of observation. Since we see both in plane and out of plane components of motion, we can rule out surface diffusion. Source data are provided as a Source Data file.

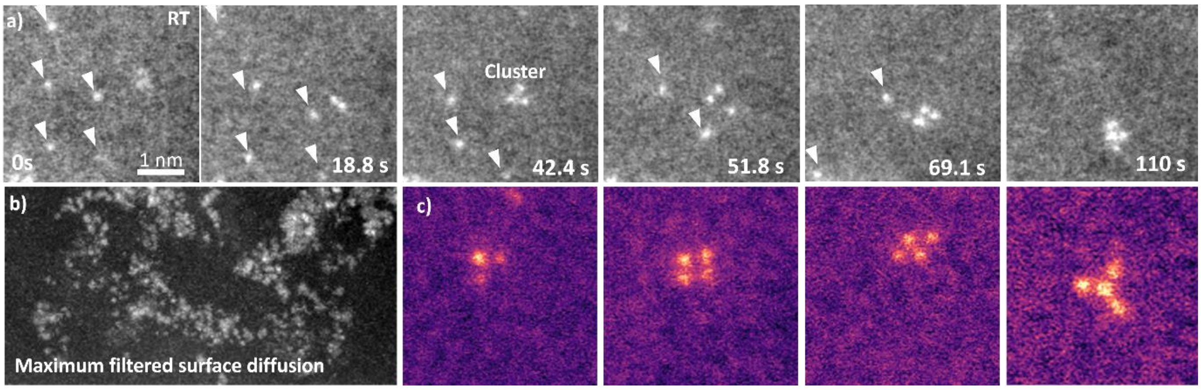

Supplementary Figure 7: Surface diffusion and cluster formation. a) Isolated atoms on a bare SiN substrate diffusing at room temperature. When atoms meet, they tend to form clusters. b) Maximum filtered image series showing random diffusion paths at room temperature not following a lattice. c) Metal cluster made of 4 atoms merged via surface diffusion, switching between different polymorphic forms.

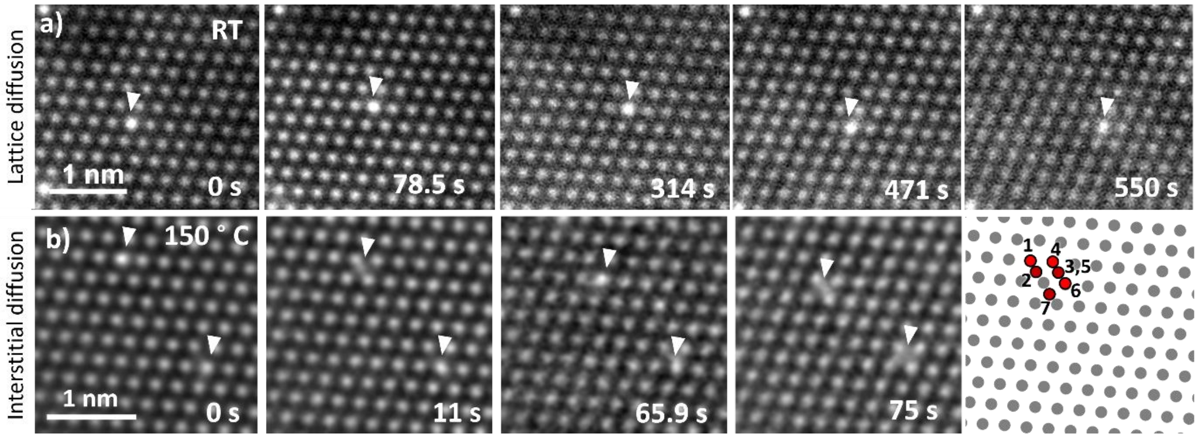

Supplementary Figure 8: Diffusion of tungsten atoms in aluminium. a) Exemplary diffusive motion of a tungsten atom in Al at room temperature via vacancy mediated lattice diffusion. b) Diffusive motion of an impurity at slightly elevated temperatures showing interstitial (or interstitialcy) type diffusion within the lattice. See supplementary movies 3 and 4 for the whole movies.

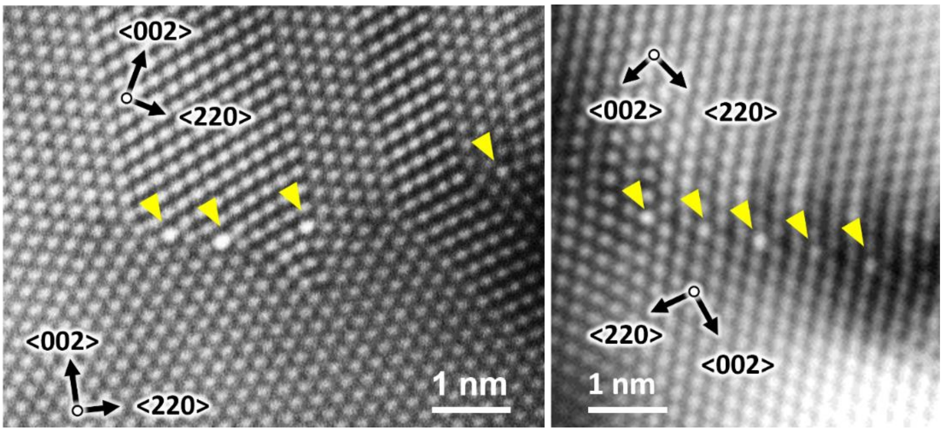

Supplementary Figure 9: Segregation effects at grain boundaries. Instead of seeing accelerated diffusion along all grain boundaries, in some cases we see segregation at grain boundaries and therefore slower

diffusion. In these instances, it is energetically favourable for impurity atoms to stay at specific sites at grain boundaries and not diffuse further.

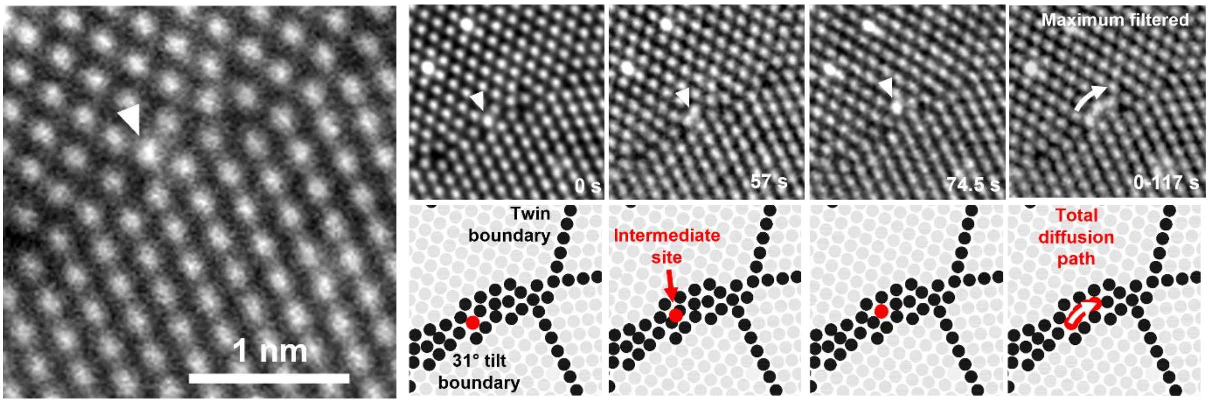

Supplementary Figure 10: Grain boundary diffusion using GB interstitial sites. a) Impurity at a high angle 110 tilt boundary. b) Impurity atom diffusion along the grain boundary at elevated temperatures. Throughout the movement of the atom, it makes use of grain boundary interstitial sites.

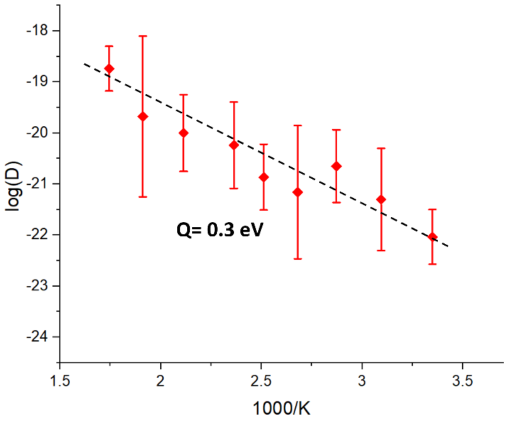

Supplementary Figure 11: Arrhenius plot of tungsten diffusion in aluminum. The activation energy is as low as 0.3 eV which can be attributed to electron beam effects. As a consequence, there is already noticeable diffusion at room temperature. The error bars represent the standard error. Source data are provided as a Source Data file.

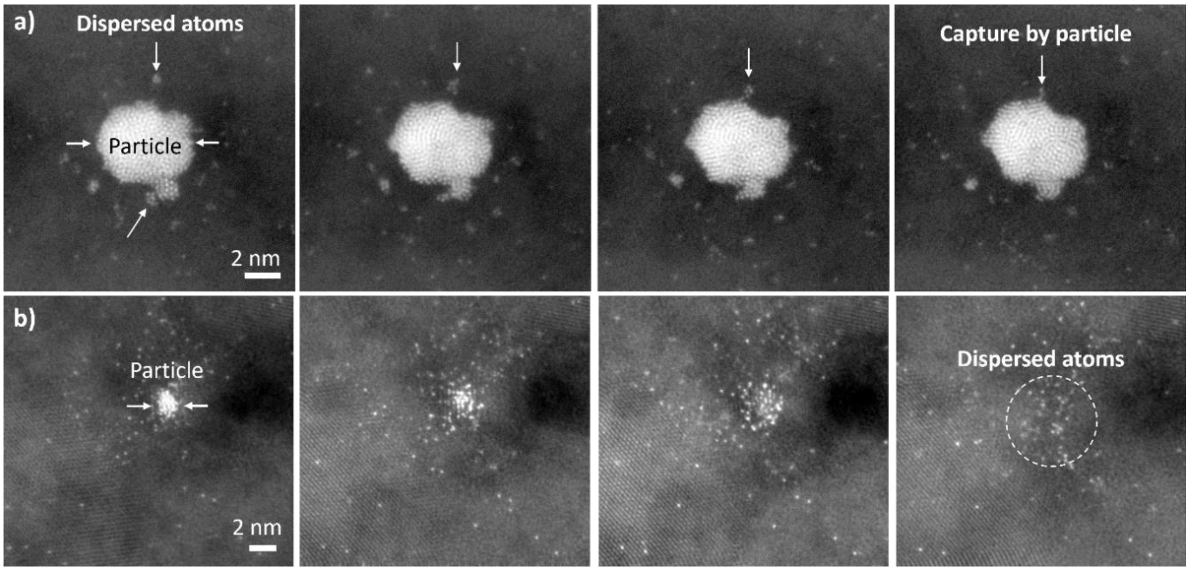

Supplementary Figure 12: Atom dynamics near nanoparticles. a) Bigger particle that acts as a sink for dispersed atoms that are captured upon coming into contact with the particle. b) Small particle that is unstable and dissolves during heating. The atoms disperse into the surrounding area.
