# Supplementary material for: Atomic scale volume and grain boundary diffusion elucidated by in situ STEM
Source: Nat Commun. 2023 Nov 22;14:7601. doi: 10.1038/s41467-023-43103-7 (PMC10663537; doi:10.1038/s41467-023-43103-7)
Supplement: Supplementary file 3 — Description of Additional Supplementary Files [file 41467_2023_43103_MOESM3_ESM.pdf]

Description of Additional Supplementary Files

Supplementary Movie 1
Description: This movie shows the volume diffusion behavior of several tungsten atoms inside a copper matrix at 385 °C. This movie corresponds to Figure 1 c in the main text. The movie (like all other movies) has been recorded using high resolution scanning transmission electron microscopy.

Supplementary Movie 2
Description: In this movie the diffusive trajectory of a single tungsten atom in a copper lattice is shown. The atom hops from lattice site to lattice site in a random walk. This movie corresponds to Figure 2 in the main text.

Supplementary Movie 3
Description: This movie shows diffusion at 450 ° C illustrating the acceleration of the atomic motion with elevated temperatures.

Supplementary Movie 4
Description: In this movie the diffusive motion of a single tungsten atom inside aluminum is shown. The atom moves via vacancy mediated jumps from lattice site to lattice site.

Supplementary Movie 5
Description: The diffusive behavior of tungsten atoms in aluminum moving via an interstitial type of mechanism is shown in this movie.

Supplementary Movie 6
Description: This movie shows diffusion along a coherent twin boundary in copper at 350 °C. This movie corresponds to Figure 3 a.

Supplementary Movie 7
Description: In this movie grain boundary diffusion at 250 °C is shown. This movie corresponds to Figure 3 a.
