# Supplementary figures and images for: Atomic scale volume and grain boundary diffusion elucidated by in situ STEM
Source: Nat Commun. 2023 Nov 22;14:7601. doi: 10.1038/s41467-023-43103-7 (PMC10663537; doi:10.1038/s41467-023-43103-7)

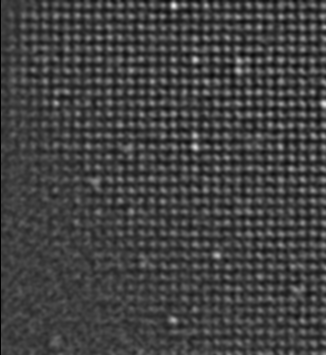

Supplement: Supplementary file 4 — Supplementary Movie 1 [file 41467_2023_43103_MOESM4_ESM.gif]

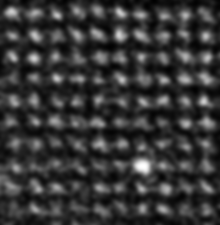

Supplement: Supplementary file 5 — Supplementary Movie 2 [file 41467_2023_43103_MOESM5_ESM.gif]

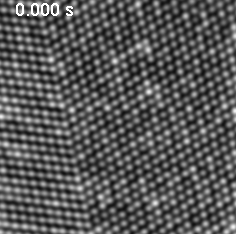

Supplement: Supplementary file 6 — Supplementary Movie 3 [file 41467_2023_43103_MOESM6_ESM.gif]

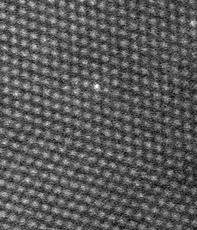

Supplement: Supplementary file 7 — Supplementary Movie 4 [file 41467_2023_43103_MOESM7_ESM.gif]

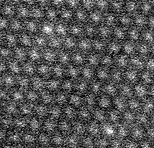

Supplement: Supplementary file 8 — Supplementary Movie 5 [file 41467_2023_43103_MOESM8_ESM.gif]

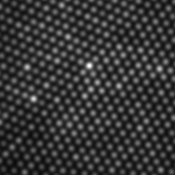

Supplement: Supplementary file 9 — Supplementary Movie 6 [file 41467_2023_43103_MOESM9_ESM.gif]

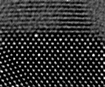

Supplement: Supplementary file 10 — Supplementary Movie 7 [file 41467_2023_43103_MOESM10_ESM.gif]
